# Management of Sarcoidosis, a Selection of Topical Items Updating

**DOI:** 10.3390/jcm9103220

**Published:** 2020-10-07

**Authors:** Dominique Valeyre, Jean-François Bernaudin

**Affiliations:** 1 UMR INSERM 1272 Université Sorbonne Paris Nord, 93000 Bobigny, France; jean-francois.bernaudin@sorbonne-universite.fr; 2 APHP Hôpital Avicenne, 93000 Bobigny, France; 3 Groupe Hospitalier Paris-Saint Joseph, 75014 Paris, France; 4 Faculté de Médecine, Sorbonne Université, 75013 Paris, France

First of all, we would like to thank all the authors for their contribution and the editorial staff who enabled the achievement of this “Management of Sarcoidosis: Challenges and Solutions” Special Issue. This issue covers a selection of hot questions on sarcoidosis pathogenesis and on everyday clinical practice in sarcoidosis. This issue is more an impressionist view of sarcoidosis than an exhaustive one, as significant progress was made both in the understanding of the pathogenesis and in diagnostic and therapeutic approaches—particularly in terms of changes in new concepts concerning genetics and auto-immunity, and a more comprehensive approach to the care. Therefore, the issue contains very didactic and practical general reviews, as well as original articles focused on little-known or mutating topics.

Sarcoidosis is a disease with a large phenotypic variability due to the complex combination of genetic susceptibility, immune networks and still unknown infectious and/or environmental causal agents. 

One way to penetrate the still opaque world of this systemic disease is in the development of selected models with a high level of confidence, which is Besnard and Jeny’s review’s objective [1]. As they recall, “models should aim to be isomorphic (replication of pathological and histopathological features), homologous (identification of the pathogenic mechanisms), and predictive (testing of the efficacy and toxicity of potential novel therapeutic strategies)”. Their review covers what should be expected and what has to date been gained from models in sarcoidosis initiated several years ago, with various degrees of success due to the disease’s complexity, its unknown causes, the various phenotypic presentations and the multiple genetic factors involved, as reviewed by Calender et al. in this issue [2]. We will pick up three examples of that have been recently added to the dissection of pathogenic mechanisms in sarcoidosis using models. Crouser et al. developed an in vitro granuloma-like model from the peripheral blood mononuclear cells of sarcoidosis patients in the presence of *Mycobacterium tuberculosis* (PPD)-coated polystyrene beads. This model makes it possible to have access to the initial cellular and molecular events in granuloma formation [3]. The second example is the model of transgenic mice with the deletion of the inhibitor tuberous sclerosis complex 2 (Tsc2) in the myeloid lineage spontaneously developing sarcoid-like granulomas [4]. As a consequence of these findings, the activation of the Mammalian Target of Rapamycin (mTOR) pathway was identified in patients’ granulomas, confirmed by genetic studies, and the use of mTOR inhibitors might become a new treatment option for sarcoidosis that is difficult to treat, if trials confirm this point [5,6]. The third example is the highlighting of the NLRP3-inflammasome pathway by Huppertz et al., thanks to cell investigation in patients and reinforced by an experimental model in transgenic mice [7]. Consecutively, a role for IL-1β, which recently regained interest in part due to the molecular delineation of the inflammasome pathway, was suggested in sarcoidosis by multiple studies almost 30 years ago, but has been quite overlooked since [8]. If the intersection of granulomatosis and autoinflammatory diseases is considered a rare occurrence, the field of autoinflammatory disorders has expanded significantly in the last decade, and the possibility of considering at least in part sarcoidosis as one of the polygenic autoinflammatory diseases is reinforced [9]. Of the three significant key cellular pathways activated in sarcoidosis brought to light within the last 3 years (the mTOR, the Janus Kinase/signal transducers and activators of transcription (JAK/STAT), and the NLRP3 inflammasome pathways) two were based on investigations of patients’ samples, associated with experimental models in mice [10].

Thanks to the development and mastery of innovative tools, significant pieces of genetics in the sarcoidosis jigsaw puzzle are added on a regular basis, which is clearly and extensively described by Calender et al. [2]. A recent Swedish retrospective case-control family study estimated a 39% mean heritability in sarcoidosis, which highlights the major role of genetics in this “Nature and nurture” disease [11]. As detailed in the various contributions to this Special Issue of the journal, the heterogeneity of clinical phenotypes, either upon presentation or during evolution, is a major argument for the widespread distribution of gene variants involved in the pathogenesis background of sarcoidosis. The authors provide in this review a thesaurus of the main genes found to be at least in part involved, classified according to the methods used Genome Wide Association Study (GWAS); Whole Exome Sequencing (WES); Next Generation Sequencing (NGS)], and the discussion of their biological and clinical significance is really informative. Through their personal research, Calender and colleagues have brought to light variants of genes involved in autophagy and in the mTOR and RAC1 pathways, which are in line with clinical and experimental observations [5,12]. If the identification of cells expressing these genes, and impaired by their variants, remains yet to be specified, disentangling the complexity of the sarcoidosis skein is in progress. 

Autoimmunity features observed in sarcoidosis have been observed for a long time, but they raise the hypothesis of an underlying autoimmune disease that co-occurs with sarcoidosis [13], or a role of sarcoidosis per se in the development of autoimmune diseases [14]. This debate has been illustrated by the Mahévas et al. study on the association of sarcoidosis and immune thrombocytopenia [15]. The report of auto-antibodies functional against Negative Elongation Factor E (NELF-E) in sarcoidosis by Baerlecken et al. makes an additional significant contribution to the discussion of the “nature” of sarcoidosis [16]. J Grunewald and his team deserve the credit for demonstrating that auto-immunity, especially towards vimentin as an autoantigen, is a component of sarcoidosis as a disease [17,18]. Vimentin was identified as a major antigen in the Kveim test substance [19], and was detected in asteroid bodies of sarcoidosis granulomas [20]. Baerlecken et al. identified in 35% of sarcoidosis (vs. 7% in controls, healthy patients or patients with auto-immune diseases) the Negative Elongation Factor E (NELF-E) as another auto-antigen [16]. Moreover, they found an association of the frequency of NELF-E antibody detection with lung parenchymal involvement. The authors emphasize that NELF-E is not expressed as an extracellular antigen, but likely becomes an antigen once exposed within inflamed and necrotic tissue. NELF-E is an elongation factor involved in DNA repair, and its link with sarcoidosis remains unclear [21]. NELF-E auto-antibodies could only be a bystander phenomenon. However, noteworthy preliminary unpublished data from the same group suggests that NELF-E auto-antibodies may develop because of a mimicry between a certain part of the amino acid sequence of NELF-E and *Mycobacteroides abscessus* or other bacteria. This noteworthy contribution from Baerlecken et al. reinforces the discussion about the classification of sarcoidosis among the vast spectrum of immune or inflammatory systemic diseases, which has always been a puzzle—immune granulomatous disease (association of innate and adaptative immunity; activation and typical granulomas), auto-inflammatory disease (inflammasome activation) or auto-immune disease (humoral and/or immune recognition of autoantigens) [16]? Or, closer to reality, a polygenic auto-inflammatory disease with autoimmune features [9,22]? 

To end with the pathogenic domain of the issue, McClain Caldwell et al. contributed a research paper opening a new field in sarcoidosis treatment, focused on the potential benefits of stem-cell therapy [23]. It should be noted that the idea has been in the air for a few years, as previously addressed by Baughman et al. in a small phase I trial, wherein they intravenously injected placental mesenchymal-like cells into four patients with chronic sarcoidosis, then followed them for two years. Two of the four patients had significant improvements [24]. In the same vein, as activated macrophages are major players [25,26], McClain Caldwell et al. hypothesized that human MSCs may reprogram alveolar macrophages from patients with sarcoidosis, and therefore could reverse the disease. In fact, unselected BAL cells, mainly macrophages, from sarcoidosis patients cocultured with mesenchymal stromal cells from healthy adults showed a switch from a pro-inflammatory to an anti-inflammatory pattern. We thought these preliminary results worthy of being published in this issue, as mesenchymal stem-cell therapy may in the long term potentially reinforce the treatment armamentarium in severe pulmonary sarcoidosis.

In the area of clinical knowledge of sarcoidosis, classical reviews are still not outdated [27,28,29]. However, this knowledge is continuously proceeding by small touches, as illustrated by Catharina Moor’s paper, the main interest of which is to give a global picture of care of sarcoidosis patients, not limited to treatment via anti-sarcoidosis drugs but addressing all aspects of the disease, from clinical to economic and social life, thanks to the use of an ABCDE model (assess/backing/complaints and comorbidities/disease-modifying treatment/extrapulmonary specialists), without minimizing the unresolved questions concerning both the cause and best medical treatment of sarcoidosis [30,31]. Apart from well-known severe complications causing organ dysfunction and premature death [32,33], Quality of life (QOL) impairment, notably due to fatigue, depression, cognitive impairment, small-nerve neuropathy, familial and occupational life or due to reduced income, which have been insufficiently taken into account for a long time, are better recognized, as is the impact on familial partners. Sarcoidosis is a multifaceted disease with variable presentations according to epidemiology, organs involved, duration and severity, often associated with impaired quality of life due to symptoms, para-sarcoidosis syndromes and treatment adverse events. A comprehensive care is particularly indicated [30]. Therapeutic decisions need to be well-balanced, taking into account not only morbidity induced with organ dysfunction and vital risks but also patients’ expectations, particularly concerning QOL and symptoms, as shown by a recent study [34]. As the drugs’ adverse effects, now better recognized, may be more or less tolerated and accepted by patients, a very important point is the treatment adherence, and physicians have to be warned of the efficacy/safety balance of treatments [35]. Schematically, there are several kinds of treatment, using disease-modifying drugs or supportive measures. In most patients, pulmonary manifestations are at the forefront, but often extrapulmonary ones need to be taken into account, with multidisciplinary discussion [30]. There are still many unexplored points, such as comparisons between different regimen doses and even different molecules as first-line treatments, while corticosteroid remains for now the only recommended initial treatment. However, some studies suggest that using lower doses than those usually recommended might be sufficient, with lower adverse effects [36,37]. The optimal duration of treatment, conditioned by the risk of relapses, is not well determined. Current pharmacological treatment is directed towards the dysregulation of the immune system, acting as a disease-modifying treatment, and is mainly based on three lines of molecules, these being respectively corticosteroids, immune-suppressive drugs (such as low-dose methotrexate and azathioprine) and eventually anti-TNFα drugs. The use of non-disease-modifying treatments, for example implantable cardiac devices or substitutive hormone therapy, may be essential for survival [38]. 

Jain et al. underscored the difficulty of the diagnosis, with often a significant delay, and most encounter comorbidities [39]. Concerning the diagnosis, the importance of expert clinical discretion, as mentioned, is crucial. Despite limitations, biomarkers are the object of an interesting focus, allowing us to classify them according to various criteria, namely diagnosis, prognosis and severity.

As underscored by Keijsers, ^18F^FDG-PET is a very efficient biomarker for determining treatable inflammatory lesions in symptomatic patients, particularly those with normal serological inflammation, like serum angiotensin-converting enzyme and serum sIL2R [40]. ^18F^FDG-PET has been indicated in three clinical contexts concerning patients with confirmed sarcoidosis, as follows: (i) patients with pulmonary fibrosis, for whom the expected beneficial treatment effect on Forced Vital Capacity can be predicted thanks to lung inflammation assessed by lung FDG uptake; (ii) patients with cardiac sarcoidosis for whom, subject to the patients’ diet before ^18F^FDG-PET being correct, a left ventricular ejection fraction gain under treatment might be expected thanks to cardiac FDG uptake; and (iii) patients with persistent disabling symptoms. Otherwise, for patients with suspected extrapulmonary sarcoidosis difficult to diagnose, such as isolated uveitis of unknown cause, central nervous system or cardiac manifestations with normal thorax CT, the evidence of ^18F^FDG uptake in both hilar and mediastinal lymph-nodes can usefully guide the diagnosis. 

Advanced pulmonary sarcoidosis is the main cause of dangerous sarcoidosis. In their paper, Sawahata et al. had the original idea of comparing the thorax CT in patients with supplemental oxygen need with a previous one done several years before for a better understanding of the radiological findings’ significance [41]. They proved that in many cases, what resembled honeycombing was actually bronchiectasis. Nevertheless, it is important to underscore that a “UIP pattern” remains possible in sarcoidosis, particularly when located at the lung bases, raising the question of whether this aspect stems from a coincidence of two distinct diseases—sarcoidosis and then idiopathic pulmonary fibrosis (IPF)—or the particular evolution of a lone sarcoidosis in patients with a predisposing genetic background [42]. However, to date, there have been too few studies devoted to the lungs in far-advanced sarcoidosis. Large studies might bring to light different lung phenotypic profiles with more or less severe pulmonary hypertension, pulmonary fibrosis and/or an air-flow obstructive pattern, which will need comprehensive investigation, involving not only imaging but also pulmonary function testing and right heart catheterization.

Pediatric sarcoidosis is an extremely rare disease with a 0.6–1.02/100,000 estimated incidence, but in the absence of international registers, the disease is probably under-reported [43,44]. Chauveau et al.’s report on child–adult transition in sarcoidosis is the first one on this topic, based on a large study [45]. Around half of the patients were cured before being adults, the other half having a very long disease and most of them needing a long-duration treatment. Interestingly, between a quarter and a fifth of children meeting the so-called “danger sarcoidosis” criteria defined for adults will recover, probably because their condition may be reversible, with high CPI (Composite Physiological Index) but not pulmonary fibrosis or pulmonary hypertension, while other cases, not initially severe, will get a dangerous sarcoidosis at adult age due to pulmonary fibrosis, pulmonary hypertension or extrapulmonary severe organ involvement. A very important point is to offer better longitudinal care, avoiding destabilizing gaps between the pediatric and the adult management of the chronic disease.

Evidencing non-caseating granulomas on histopathology is very important for diagnosing sarcoidosis. Despite many original articles and reviews focusing on endobronchial ultrasound-guided transbronchial needle aspiration (EBUS), the particularly well-written paper by Pedro et al. offers a means of getting very clear and accurate data concerning the advantages and the pitfalls of every endoscopic technique in sarcoidosis, and the impacts of many technical factors [46]. EBUS has a superiority in diagnosis over conventional bronchoscopic modalities for radiographic stage I and II sarcoidosis, and has been recommended as the initial mediastinal and/or hilar lymph node sampling procedure in the 2020 official ATS clinical practice guideline [47]. However, conventional bronchoscopic techniques, like combined endobronchial and transbronchial lung biopsy, remain more recommended for other radiographic stages. The indication for transbronchial lung cryobiopsy is restricted to patients with suspected pulmonary sarcoidosis and inconclusive transbronchial lung biopsy [48]. 

In conclusion, while all the numerous questions raised by sarcoidosis could not have been included in the register of this Special Issue, such as for example the potential role of the microbiota in sarcoidosis [49], or a new attempt to distinguish clinical phenotypic subgroups (allowing for searching for links with genetic or environmental factors [50,51]), we think that readers will find significant information for their research or everyday practice.
